# βPix is a New Player in Renal Physiology

**DOI:** 10.3389/fphys.2012.00268

**Published:** 2012-07-20

**Authors:** Kirk L. Hamilton, Alan C. Pao

**Affiliations:** ^1^Department of Physiology, Otago School of Medical Sciences, University of OtagoDunedin, New Zealand; ^2^Division of Nephrology, Department of Medicine, Stanford UniversityStanford, CA, USA

Small G proteins (small GTP-binding proteins; GTPases) are low molecular weight proteins that play major regulatory roles in numerous biological pathways including signal transduction, regulation of cellular polarity, actin and microtubule dynamics, gene transcription, cell cycle progression, and vascular transport pathways (Etienne-Manneville and Hall, 2002). Rho GTPases are one of the group of GTPases, which include RhoA, Rac1, and Cdc42 (Etienne-Manneville and Hall, 2002; Ory and Gasman, 2011). These small monomeric GTPases serve as molecular switches by cycling between an “active state” (bound to GTP) and an “inactive state” (bound to GDP) and by hydrolyzing GTP to GDP (Etienne-Manneville and Hall, 2002; Ory and Gasman, 2011). Guanine nucleotide exchange factors (GEFs) are responsible for the recruitment and activation of Rho GTPases at the cell membrane, whereas GTPase activating proteins (GAPs) inactivate the Rho GTPases (Ory and Gasman, 2011).

The focus of this Commentary is to highlight the recent review article by Staruschenko and Sorokin (2012) published in *Frontiers of Physiology* in which they have provided a brief background of the GEF βPix, but more importantly, they have reviewed the recent and very exciting roles of βPix in kidney physiology. βPix [p21-activated kinase (PAK)-interacting exchange factor β] is a GEF that modulates Rac1 and Cdc42 (Guilluy et al., 2011). As far as we can determine, there has only been a handful of reviews that address the biology and function of βPix and the related GEF αPix (Bagrodia and Cerione, 1999; Rosenberger and Kutsche, 2006; Frank and Hansen, 2008; Schlenker and Rittinger, 2009; Momboisse et al., 2010).

For those readers unfamiliar with β-Pix (*ARHGEF 7*), this protein has had a number of previous names including COOL1, KIAA0142, P50BP, P85, P85SPR, PAK3, and PixB (HUGO Gene Nomenclature Committee; http://www.genenames.org/data/hgnc_data.php?hgnc_id=15607). Oh et al. (1997) originally demonstrated that p85SPR [Src Homology 3 (SH3) domain containing proline-rich protein], now known as βPix, interacted with areas of focal adhesion, suggesting a role for βPix in cytoskeletal function. Shortly thereafter, Manser et al. (1998) reported the binding of βPix (and αPix) to PAK1. Further, Bagrodia et al. (1998) identified βPix (named p85Cool-1) and a smaller alterative splice variant (p50Cool-1) as two proteins that facilitated interactions between PAK and DBL homology (DH) and pleckstrin homology (PH) domains. Finally, Koh et al. (2001) reported an isoform of βPix designated β_2_Pix; that isoform contained a serine-rich region not found in the original βPix protein (which is now designated as β_1_Pix-a, Kim et al., 2000) nor the β_1_Pix-b and β_1_Pix-c isoforms (Oh et al., 1997; Kim et al., 2000). The structure and functional domains of β_1_Pix are provided in Figure 1.

**Figure 1:**
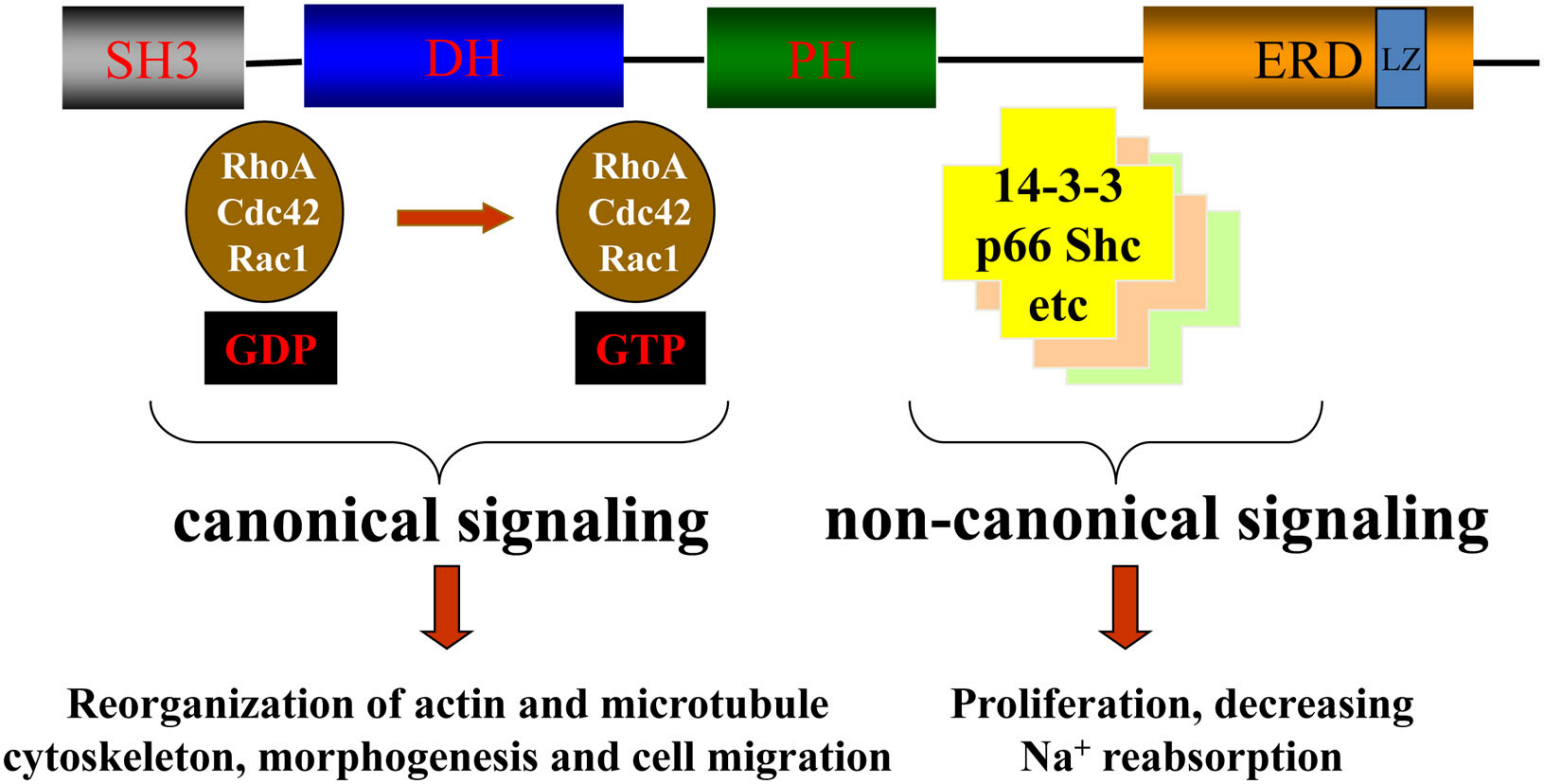
**Domain structure of βPix and the canonical and non-canonical signaling**. βPix is 647 amino acids long (Staruschenko and Sorokin, 2012). Note, the amino length of βPix is variable depending upon the species and possible isoforms within a species. βPix is composed of a series of domains that interact with various molecules. These domains include the SH (Src Homology 3) domain, DH (DBL homology) domain, PH (pleckstrin homology) domain, GIT1 (G-protein coupled receptor kinase-interacting binding motif, not shown) domain, ERD (glutamic rich regions) domain, and the LZ (leucine zipper) domain. Staruschenko and Sorokin (2012) have described the functions of these domains in which various domains are important with specific interactions of βPix which interact at areas of focal adhesion (SH), mediating guanine nucleotide exchanges on some Rho family GTPases (DH), binding to phosphatidylinositol lipids and proteins (PH), and dimerization of the Pix molecules (LZ). βPix plays a number of roles that are dependent upon its GEF functions (canonical signaling) and its scaffolding functions (non-canonical signaling). This figure was used with permission from the authors (Staruschenko and Sorokin, 2012) and Frontiers of Physiology.

There are a number of functions of β_1_-Pix. Staruschenko and Sorokin (2012) describe that β_1_Pix participates in both canonical and non-canonical signaling pathways involved in various cellular functions (see Figure 1). The canonical signaling of β_1_Pix results from its GEF activity, which activates Rac1 and Cdc42, and regulates various cellular functions including cytoskeletal reorganization, morphogenesis, and cell migration (Figure 1). β_1_Pix also exhibits non-canonical activities in which it serves as a scaffolding protein in some signaling pathways (Pavlov et al., 2010).

Staruschenko and Sorokin (2012) also provide an overview of the expression of βPix in the kidney and the various roles of βPix in kidney function. Recently, βPix expression has been detected in mesangial cells, podocytes, cortical collecting ducts, and localized vessels and vascular smooth muscle cells of the rat kidney and in a number of nephron segment-specific derived cell lines (antibodies against βPix were unable to discriminate between the β_1_Pix and β_2_Pix isoforms, Pavlov et al., 2010). These findings set the stage for unraveling the roles of βPix in renal physiology, which is presented under four categories (Staruschenko and Sorokin, 2012): (i) regulation of ion transport, (ii) regulation of glomerular function, (iii) regulation of urothelial signaling, and (iv) complexity of βPix signaling in the kidney.

One of the most exciting advances in our understanding of β_1_Pix function in the kidney involves the role of β_1_Pix in regulating the epithelial sodium channel (ENaC) in the cortical collecting duct. Staruschenko and colleagues (Pavlov et al., 2010) have recently demonstrated that endothelin-1 signals through β_1_Pix to decrease the number of ENaC channels in the apical cell membrane of cortical collecting duct cells. β_1_Pix negatively regulates ENaC by binding to 14-3-3 proteins and disrupting the interaction between 14-3-3 proteins and the E3 ubiquitin ligase Nedd4-2. A major regulator of ENaC, Nedd4-2 ubiquitinates cell surface ENaC, marking the channel for internalization and degradation. Since 14-3-3 proteins inhibit Nedd4-2 activity, β_1_Pix blocks 14-3-3 proteins from interacting and inhibiting Nedd4-2, thereby enabling Nedd4-2 to inhibit ENaC. Interestingly, this inhibitory effect is dependent on the role of β_1_Pix as a scaffold protein rather than a GEF.

To date, there have been no reports of any mouse models or human diseases that are associated with βPix deficiency or dysfunction. There are, however, studies that implicate βPix over-expression in human breast cancer tissue, suggesting that βPix plays a significant role in controlling cell proliferation and carcinogenesis and may be a potential marker of malignant disease (Ahn et al., 2003). In future studies, the relative contribution of various βPix functions in the kidney will need to be confirmed *in vivo*.

## Final Thoughts

The review paper by Staruschenko and Sorokin (2012) is very timely as the role of βPix in a number of tissues is still emerging, especially within the kidney. Certainly as βPix knock-out mice models are generated, additional new and exciting role(s) of βPix will be clearly demonstrated. Additionally, experiments that isolate the canonical and non-canonical pathways by which βPix operates will define very specific functions of βPix within the kidney and possibly lead to the development of novel treatment strategies for renal disease.
